# On Certain Fermentative Processes in the Alimentary Canal, and the Micro-Organisms by Which They Are Produced

**Published:** 1886-02

**Authors:** 

**Affiliations:** Berlin


### ON CERTAIN FERMENTATIVE PROCESSES IN THE ALIMENTARY CANAL, AND THE MICRO-ORGANISMS BY WHICH THEY ARE PRODUCED.

BY PROF. DR. MILLER, BERLIN.

Of the three divisions of the alimentary canal—mouth, stomach and intestines—the mouth furnishes, under many circumstances, the best conditions for the growth of micro-organisms. The juices which, in the stomach and upper part of the small intestines, oppose a powerful hindrance to the development of bacteria, are wanting in the human mouth, while at the same time the most diverse nutritive materials are there provided with free access of air and favorable temperature. The number of fungi which may develop in the human mouth is therefore very large, and limited almost alone by the concurrence of the different forms. The largest number which I have found in any one mouth at the same time is eleven, not including the well-known *Leptothrix buccalis*, *Spirochœte dentium*, and *Vibrio buccalis*, which no one has as yet succeeded in cultivating. The fermentative and putrefactive changes in the human mouth, corresponding to the large number of fungi and the favorable conditions for their development, acquire, under certain circumstances, a high if not a dangerous intensity. In proof of this I need only to call attention to the exceedingly disagreeable, stinking odor which comes from the mouth in cases of stomatitis ulcerosa, of gangrene of the dental pulp, etc., etc., or to the fact that a slight scratch on the finger from a root in an unhealthy mouth may produce the most dangerous symptoms of blood poisoning.

Of twenty-five different kinds of bacteria which I have isolated from the secretions of the human mouth, twelve are cocci and thirteen bacilli or bacteria. It was not possible in all cases to make a distinction between bacilli and bacteria, since many kinds produce at the same time long rods (bacilli), and short rods (bacteria).

Twelve of the mouth-bacteria I found again in the fæces, and eight in the contents of the stomach. In the latter case the material for the investigations was furnished by a gentleman who could evacuate his stomach at will, an hour or two after partaking of a small quantity of fruit, particularly strawberries. Each time the oral cavity was carefully cleaned and sterilized with sublimate, one to one thousand, in order to prevent the intermixture of fungi from the mouth. That the micro-organisms really came from the stomach, and not from the mouth or œsophagus, could readily be seen by their large numbers. I have satisfied myself by repeated experiments, that in plate cultures from the saliva of a mouth which has been treated as stated above, very few, or no colonies at all, will be developed, while in the cultures from the contents of the stomach, a small drop in 5.0 cc. of gelatine would produce colonies so numerous that the separate ones were indistinguishable. Again, all organisms which were represented by only a few colonies were excluded.

It is, perhaps, allowable to take for granted that all the stomach bacteria enter the stomach along with the food; it is much less probable that they find entrance from the duodenum, although the possibility cannot be entirely excluded. It would not be permissible to assume that the intestinal bacteria all came from the stomach, since an entrance per anum is not to be overlooked, and since the stomach is supposed to present an impassable barrier for most micro-organisms. The results obtained from the experiments to be described indicate, however, that the latter is not the case, but that any fungus under a variety of conditions may readily pass the stomach without losing its power of development. The resistance of many micro organisms to the action of the gastric juice may be readily seen from the following experiment:

About three hours after a moderate meal, 70 cc. of the contents of the stomach were evacuated and placed in the incubator under exclusion of air germs. Three hours later, by means of plate cultures, I found three kinds of bacteria, two yeast and one mould fungus, and after ten hours one bacterium form, and the yeast and mould fungi still living. Not till after the lapse of fourteen hours were all bacteria missing. The Blastomycetes and Hyphomycetes were naturally not affected by the acid. The fact that a fungus in an artificial gastric juice may lose its vitality in a short time, is no proof that the same may not safely pass through the stomach, because:—

1st. The fungi which are swallowed at the beginning of a meal do not pass into a stomach filled with gastric juice, but into an empty stomach with a neutral or alkaline reaction, where free hydrochloric acid, in detectable quantities, does not appear until after the lapse of one-half to one and one-half hours.

2d. The fungi are often imbedded in solid particles of food, thus escaping for a while the action of the juice.

3d. Liquid substances do not remain long in the stomach, but soon pass into the duodenum, and carry with them the fungi before any considerable quantity of gastric juice has been secreted. In a case of fistula in the upper part of the duodenum, Busch saw the first portions of food appear fifteen or twenty minutes after the beginning of the meal (*vid.* Hoppe Seyler, Phys. Chem. 326). In case of soft or liquid food, the transit may begin still sooner. The experiments of Watson, Cheyne and others, in connection with this subject, do not appear to me to be conclusive because made under conditions too little similar to those actually present in the stomach. Cheyne added material containing micro-organisms to a comparatively large quantity of artificial gastric juice, and observed that they were destroyed in a short time. Such experiments simply show that the normal gastric juice has antiseptic properties sufficiently strong to devitalize certain bacteria within a certain time. They give us, however, very little information as to the real occurrences in the stomach itself.

In order to reproduce as nearly as possible the conditions present in the normal stomach, I chewed up a quantity of bread and meat, added a small proportion of liquid (milk), and divided the mixture into portions of 26.0 cc. each. These portions were brought into small flasks, sterilized and then infected, some with a very sensitive vibrio, the others with hardy ferment-bacteria from the stomach. To each portion was now added every ten minutes 2.0 cc. of an artificial gastric juice, containing 0.4 per cent. H. cl., (1.6 per cent. H. cl. solution of sp. gr. 1,1233), so that at the end of the second hour, the mixtures contained each 0.2 per cent. H. cl., corresponding to the most active point of normal gastric digestion, which has been determined to come at about the end of the second hour or a little later, when the acidity of the stomach has reached its highest degree. Cultures made from time to time during the course of the experiments from the different flasks showed that the least resistive of the micro-organisms experimented with retained their vitality till the end of the third half-hour, and sometimes longer, and that the less sensitive ferment-bacteria showed no diminution in number at the end of the experiment, and, in many cases, some of them were still found six to eight hours later. Inasmuch as portions of food pass into the duodenum in less than half an hour after taking food, these experiments seem to indicate that any micro-organism swallowed at the beginning of a meal might readily pass through the stomach alive. The case is somewhat different, however, if the fungi are swallowed when the digestion is at its most active stage (second and third hour). Experiments representing this stage of digestion showed that very sensitive putrefactive bacteria may thus be destroyed within ten minutes, while the hardiest ferment-bacteria may resist for hours.

From these experiments the conclusion appears warranted that all fungi which are swallowed at the beginning of a meal may pass alive into the intestines, while of such as are swallowed in the second or third hour, only those which are less sensitive to the action of acids retain their vitality. If we furthermore take into consideration the various and numerous affections in which the quantity of the gastric juice or its percentage of H. cl. is abnormally small, it will appear as though the stomach affords almost no protection whatever against the entrance of pathogenic organisms into the intestinal canal, and that the condition of the intestines themselves must be looked upon as the factor which determines whether a pathogenic micro-organism, which has entered the alimentary tract, shall or shall not come to development or manifest its characteristic action in the intestines. We have an exact proof that such is the case with the cholera bacillus. Koch found that, in order to make guinea-pigs susceptible to the cholera poison, it did not suffice to secure the passage of cholera bacilli into the duodenum by neutralizing the contents of the stomach, but that it was necessary first to induce an atonicity of the intestines themselves, with cessation of the peristaltic action.

It is worthy of mention that, although particles of food take up the acid with avidity, they nevertheless often appear to afford a certain protection to the micro-organisms. I have repeatedly observed in the plate cultures, that muscular fibres in particular were lined with colonies, while on other parts of the plate very few were to be seen.

(TO BE CONTINUED.)

